# Investigation of the *Staphylococcus aureus* GraSR Regulon Reveals Novel Links to Virulence, Stress Response and Cell Wall Signal Transduction Pathways

**DOI:** 10.1371/journal.pone.0021323

**Published:** 2011-07-01

**Authors:** Mélanie Falord, Ulrike Mäder, Aurélia Hiron, Michel Débarbouillé, Tarek Msadek

**Affiliations:** 1 Institut Pasteur, Biology of Gram-Positive Pathogens, Department of Microbiology, Paris, France; 2 CNRS, URA 2172, Paris, France; 3 Interfaculty Institute for Genetics and Functional Genomics, Department for Functional Genomics, Ernst Moritz Arndt University, Greifswald, Germany; org-nameUniversity of Liverpool, United Kingdom

## Abstract

The GraS/GraR two-component system has been shown to control cationic antimicrobial peptide (CAMP) resistance in the major human pathogen *Staphylococcus aureus*. We demonstrated that *graX*, also involved in CAMP resistance and cotranscribed with *graRS*, encodes a regulatory cofactor of the GraSR signaling pathway, effectively constituting a three-component system. We identified a highly conserved ten base pair palindromic sequence (5′ ACAAA TTTGT 3′) located upstream from GraR-regulated genes (*mprF* and the *dlt* and *vraFG* operons), which we show to be essential for transcriptional regulation by GraR and induction in response to CAMPs, suggesting it is the likely GraR binding site. Genome-based predictions and transcriptome analysis revealed several novel GraR target genes. We also found that the GraSR TCS is required for growth of *S. aureus* at high temperatures and resistance to oxidative stress. The GraSR system has previously been shown to play a role in *S. aureus* pathogenesis and we have uncovered previously unsuspected links with the AgrCA peptide quorum-sensing system controlling virulence gene expression. We also show that the GraSR TCS controls stress reponse and cell wall metabolism signal transduction pathways, sharing an extensive overlap with the WalKR regulon. This is the first report showing a role for the GraSR TCS in high temperature and oxidative stress survival and linking this system to stress response, cell wall and pathogenesis control pathways.

## Introduction

The opportunistic pathogen *Staphylococcus aureus* is both a commensal and a major Gram-positive pathogen, causing a variety of infections ranging from superficial skin abscesses to more serious diseases such as pneumonia, meningitis, endocarditis, septicemia and toxic shock syndrome [1]. The ubiquitous nature of this pathogen stems mostly from its capacity to survive a large variety of environmental conditions as well as an impressive ability to resist host innate immune defense mechanisms and produce systemic infections, often in healthy humans [2], [3]. This unique adaptive potential has made *S. aureus* one of the major causes of nosocomial infections today, compounded by the rapid emergence of multiple antibiotic-resistant strains over the past few decades [4], particularly methicillin and vancomycin-intermediate resistant strains (MRSA and VISA). Until recently, vancomycin had remained the weapon of last resort, but the recent appearance of the enterococcal *vanA* vancomycin-resistance gene cluster in *S. aureus* highlights the growing threat this bacterium poses to human health and the urgent need for developing novel therapeutic approaches [5], [6].

Cationic antimicrobial peptides (CAMPs) are an important component of host innate immunity and understanding the molecular mechanisms involved in resistance is a key factor in staphylococcal treatment research. CAMPs have both cationic and amphipathic properties and are classified according to their length and secondary structure [7]. They are produced by certain immune, skin and epithelial cells in all living kingdoms, as defenses against microbial proliferation, and many are known to act by forming pores in the cell membrane, through interactions with bacterial cell envelope components [8]. However, recent work has shown that several CAMPs, including indolicidin and colistin, can also kill by inhibiting intracellular processes such as protein and DNA synthesis as well as septum formation and division [9].

To counteract CAMP antimicrobial activity during infection, Gram-positive bacteria have developed several resistance mechanisms, including degradation, sequestration or electrostatic repulsion [10]. In *Bacillus subtilis* and related Gram-positive bacteria, D-alanylation of wall teichoic acids (WTAs) and lipoteichoic acids (LTAs), mediated by the DltABCD enzymes, as well as MprF-dependent lysylination of phosphatidylglycerol, prevent CAMP-binding by increasing the bacterial surface positive charge [10], [11].

Two-component systems (TCSs) play an important role in these mechanisms by coordinating the expression of resistance genes, when CAMPs are detected at the cell surface. TCSs are typically composed of a membrane histidine kinase (HK), acting as a signal sensor/transducer, through phosphorylation of its cognate response regulator (RR), which acts as a transcription activator or repressor. Most *S. aureus* genomes have a sophisticated arsenal of sixteen sets of environmental monitoring TCS genes, with an additional one present in the staphylococcal cassette chromosome *mec* element of MRSA strains [12]. Among these systems, the well-studied AgrCA peptide quorum-sensing TCS controls the expression of several virulence genes [13] and VraSR was shown to be responsible for resistance to cell wall-damaging compounds, including β-lactam antibiotics and some CAMPs [14].

The main regulatory pathway controlling CAMP resistance in staphylococci, however, is the GraSR (Glycopeptide Resistance Associated) TCS, aka ApsSR (Antimicrobial Peptide Sensor), which has been extensively studied over the past five years [15]–[18]. First discovered in *S*. *aureus* as a locus whose overexpression led to increased vancomycin resistance, the GraSR TCS was shown to be required for resistance of *S*. *aureus* and *S*. *epidermidis* to several CAMPs, by controlling expression of *mprF* and the *dlt* and *vraFG* operons [15]–[19]. Additionally, the first gene of the *graRS* operon encodes GraX, a protein of unknown function that also plays a role in CAMP resistance [16]–[19]. Missense mutations in the *graRS* locus have been linked to CAMP sensitivity of certain *S*. *aureus* strains [20], and the system also plays a role in biofilm formation [21], [22]. GraS was shown to play a role in survival of *S*. *epidermidis* and *S*. *aureus* within neutrophils [23] and the GraSR system has been implicated in *S*. *aureus* virulence in several experimental models [18], [24]–[26].

In this study we set out to further define the GraSR regulon in *S*. *aureus*. We identified a highly conserved palindromic sequence as the likely GraR binding site, and showed that the GraSR TCS is required for growth of *S. aureus* at high temperature and resistance to oxidative stress. Using a combination of genome-based predictions and transcriptome analysis, we revealed several novel GraR target genes as well as unsuspected links with the AgrCA and WalKR TCSs.

## Results

### GraX, GraS and GraR are required for *Staphylococcus aureus* colistin resistance

In an effort to fully define the GraSR regulon of *Staphylococcus aureus*, and determine the roles of GraXSR in colistin resistance, we constructed mutant strains ST1036 (Δ*graRS*) and ST1070 (Δ*graX*) in the *S. aureus* HG001 background [27] by removing the entire coding sequences of the genes (Δ*graRS*), or by an in-frame deletion (Δ*graX*; see Materials and Methods). GraX, predicted to be a cytoplasmic protein, presents weak similarities to sugar epimerases, and has been shown to be involved in CAMP resistance along with the GraSR TCS, but its specific role remains to be established.

Minimal inhibitory concentration (MIC) values for resistance to colistin, a bacterial CAMP, were determined by following growth in TSB at 37°C over a 12 h period, using a Biotek Synergy Microplate reader, with decreasing concentrations of colistin (Table 1). The Δ*graRS* and Δ*graX* mutants displayed acute sensitivity to colistin compared to the parental strain. However, the Δ*graX* mutant appeared to be more resistant to colistin than the Δ*graRS* mutant. We therefore analyzed *graR* expression in the Δ*graX* mutant by quantitative RT-PCR (qRT-PCR), showing that *graR* expression is increased approximately 2-fold compared to the parental strain (data not shown). This is likely through stabilization of the *graRS* transcript due to increased proximity with the operon promoter in the Δ*graX* mutant, suggesting that CAMP sensitivity of the Δ*graX* mutant may in fact be underestimated. We also observed that the Δ*graRS* and Δ*graX* mutants were highly sensitive to nisin (data not shown) as previously observed [17], [18].

**Table 1:** GraX, GraS and GraR are required for colistin resistance.

Strain	Relevant genotype	Colistin MIC (µg ml^−1^)
ST1120	HG001 pMK4-Pprot	700
ST1117	Δ*graRS* pMK4-Pprot	100
ST1116	Δ*graRS* pMK4-Pprot*graR*	700
ST1070	Δ*graX*	300

Strains were grown at 37°C in TSB with decreasing colistin concentrations. Growth was followed by measuring absorbance at 600 nm using a microtiter plate reader and MICs were determined as the colistin concentration inhibiting strain growth after 12 h. Each experiment was repeated at least three times.

In order to complement the Δ*graRS* mutant, an intact copy of the *graR* gene was introduced on a multicopy plasmid, resulting in strain ST1116 (Δ*graRS* pMK4-Pprot*-graR*). Complementation of the Δ*graRS* mutant by constitutive expression of the *graR* gene fully restored colistin resistance (Table 1). Indeed, it is well known that response regulator overexpression can complement the absence of the cognate kinase, due to its phosphorylation by other phosphate donors such as acetyl phosphate or aspecific kinase activity within the cell [28], [29].

### GraXSR do not autoregulate their own synthesis

**Figure 1:**
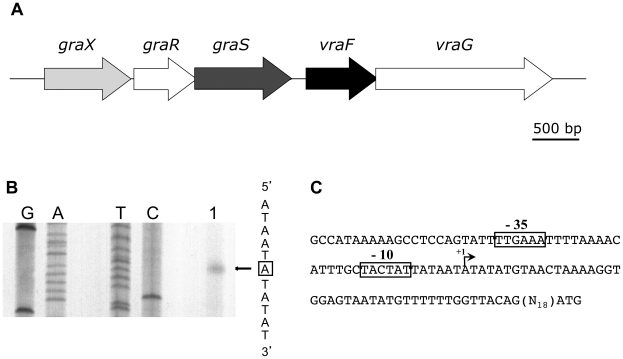
The *graXRS* operon is transcribed from a σ^A^ promoter. (A) The *graXRS*/*vraFG* locus of *S*. *aureus* HG001. (B) Primer extension analysis of *graXRS* mRNA was carried out using total RNA extracted from *S. aureus* strain HG001 during mid-exponential growth in TSB at 37°C. Primer extension experiments were performed using the *graX*-specific oligonucleotide MF63 (lane 1). The corresponding Sanger dideoxy chain termination sequencing reactions (GATC) were carried out on a PCR-generated DNA fragment fragment corresponding to the *graX* upstream region (MF62/MF63). The transcriptional start site is boxed. (C) Nucleotide sequence of the *graXRS* operon upstream region. Potential σ^A^-type -35 and −10 sequences are boxed and the transcriptional start site is labelled +1.

As shown in Fig. 1A, the *graXRS* operon is located directly upstream from the *vraFG* operon, encoding an ABC transporter [19]. To define the *graXRS* operon promoter region, we first analyzed its expression using primer extension experiments. Total RNA was extracted from strain HG001 during mid-exponential growth in TSB at 37°C and used for primer extension experiments. We identified a unique transcriptional start site in the *graXRS* promoter region (Fig. 1B), and the preceding nucleotide sequence revealed appropriately spaced potential −10 and −35 regions, sharing strong similarities with the consensus sequences of promoters recognized by the vegetative form of RNA polymerase holoenzyme, Eσ^A^ (Fig. 1C).

**Figure 2:**
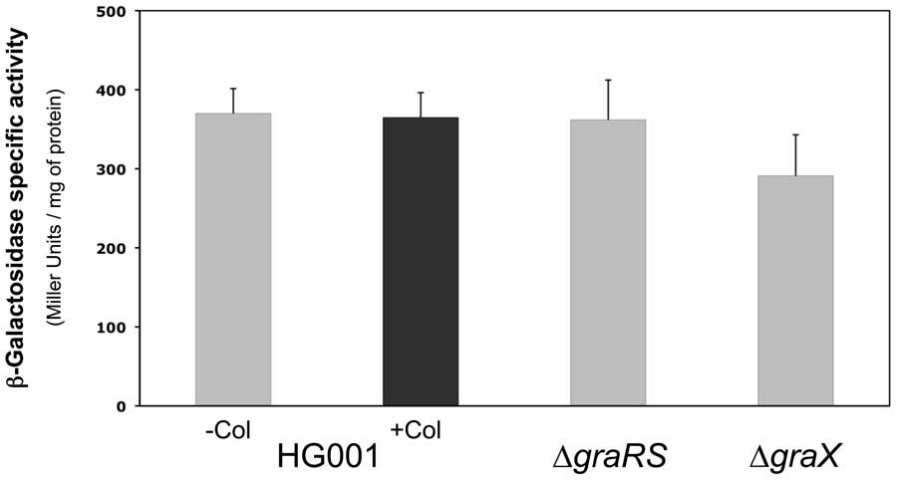
GraXSR do not control their own synthesis. Expression of the *graXRS* operon was followed using a *graX*'*-lacZ* transcriptional fusion in *S. aureus* strains HG001, ST1036 (Δ*graRS*) and ST1070 (Δ*graX*). β-Galactosidase assays were performed as described in Materials and Methods and measured during mid-exponential growth at 37°C in TSB (grey bars) or after treatment with 200 µg ml^−1^ colistin for the HG001 strain (black bar). Means and standard deviations values are presented from at least three independent experiments.

Several two-component systems are known to positively autoregulate their own synthesis, such as the VraSR cell envelope stress response and AgrCA peptide quorum-sensing virulence regulatory systems of *S*. *aureus* [13], [30]. In order to test whether this was also the case for the GraSR system, a transcriptional fusion was constructed between the *graXRS* operon upstream region and the *lacZ* gene of *E. coli* using the pSA14 plasmid (see Materials and Methods). To study *graXRS* operon expression, the *graX*'-*lacZ* fusion was introduced into strains HG001, ST1036 (Δ*graRS*) and ST1070 (Δ*graX*), and β-galactosidase activity was measured during mid-exponential growth at 37°C in TSB after a 30 mn treatment with or without 200 µg ml^−1^ colistin (Fig. 2). No significant differences in *graX*'-*lacZ* expression levels were observed between the three strains, or in the presence or absence of colistin, indicating that GraXSR do not autoregulate their own synthesis, and that their cellular levels are not induced by the presence of CAMPs such as colistin.

### Identification of potential GraR-binding sites in the promoters of known GraR regulated genes

**Figure 3:**
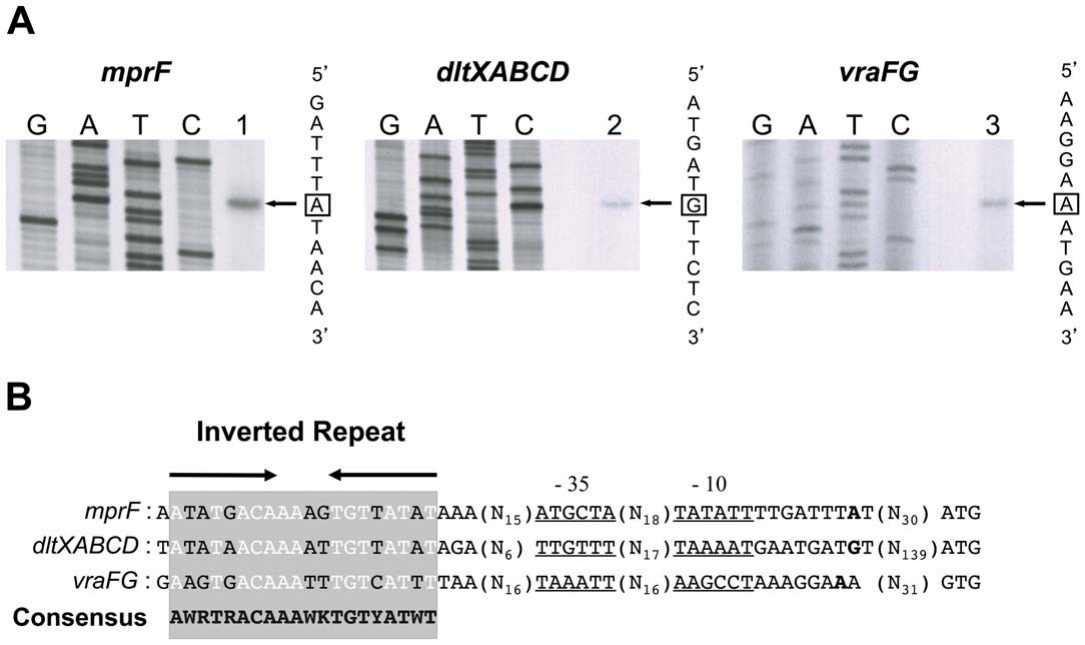
Identification of potential GraR-binding sites in the promoters of known GraR-regulated genes. (A) Primer extension analysis of *mprF*, *dltXABCD* and *vraFG* transcripts was carried out using total RNA extracted from *S. aureus* strain HG001 treated with 200 µg ml^−1^ colistin during mid-exponential growth at 37°C in TSB, using specific oligonucleotides for *mprF*, *dltX* and *vraF* (lanes 1 to 3 respectively). The corresponding Sanger dideoxy chain termination sequencing reactions (GATC) were carried out on PCR-generated DNA fragments corresponding to the respective upstream regions (see Table 5). The transcriptional start sites are boxed. (B) Alignment of the potential GraR DNA-binding sites in the *mprF*, *dltXABCD* and *vraFG* promoter regions. The potential GraR-binding site is shaded and conserved nucleotides are shown in white. Potential −35 and −10 sequences are underlined and the transcriptional start sites are indicated in bold.

Although several genes involved in CAMP resistance are known to be controlled by GraSR (*mprF*, *dlt* and *vraFG* operons), the specific nucleotide sequence constituting the GraR operator sequence remains unknown. In order to identify potential GraR-binding sites upstream from the coding regions of these genes, we first identified their transcription initiation sites through primer extension analysis. Total RNA was extracted from strain HG001 during mid-exponential growth in TSB at 37°C after treatment with 200 µg ml^−1^ colistin and used for primer extension experiments. We identified a unique transcriptional start site in the *mprF* and *vraFG* promoter regions (Fig. 3A) and two initiation sites for the *dltXABCD* operon. The first (not shown here), is located 30 bp upstream from the *dltX* (SAOUHSC_00868) translation initiation codon, and was previously identified in *S. aureus* SA113 [31] whereas the second (Fig. 3A), 110 bp further upstream, had not been reported. We identified appropriately spaced potential −10 and −35 regions upstream from all three transcription initiation sites (Fig. 3B). The *mprF* and *dltXABCD* −10 regions share strong similarities with the consensus sequence recognized by the vegetative form of RNA polymerase holoenzyme, Eσ^A^. However, all three −35 regions as well as the −10 region of the *vraFG* operon showed only weak similarities with RNA polymerase Eσ^A^ consensus promoter sequences, consistent with the existence of a positive transcriptional regulator [32].

GraR is a member of the OmpR subfamily of response regulators, with a typical winged helix-turn-helix domain [33] extending from residues 173 to 203. Although response regulators belonging to the OmpR family are known to bind to short direct repeats [34], orthologs of GraR such as VirR of *Listeria monocytogenes* and BceR of *Bacillus subtilis* were in fact shown to bind to inverted repeat sequences [35], [36]. We failed to identify any significant direct repeat sequences in the upstream regions of *mprF* and the *vraFG* and *dlt* operons. However, a global study aimed at identifying response regulator binding sites in low G+C Gram-positive bacteria [37] reported the presence of an imperfect palindromic sequence (5′ AAGTGACA-N4-TGTCATTT 3′) within the end of the *graS* coding region, upstream from the *vraFG* operon which is known to be controlled by the GraSR system [16], [17], [18], [19]. We were able to identify this palindromic sequence as also being present upstream from the −35 sequences of *mprF* and the upstream *dlt* operon promoter. The three inverted repeats are highly conserved, allowing us to align them in order to produce a potential GraR-binding site consensus sequence (Fig. 3B). In agreement with our results showing that GraXSR do not autoregulate their own synthesis, the potential GraR operator sequence is not present in the *graXRS* operon upstream promoter region.

### GraSR-dependent gene expression requires GraX, CAMPs and the consensus binding site

**Figure 4:**
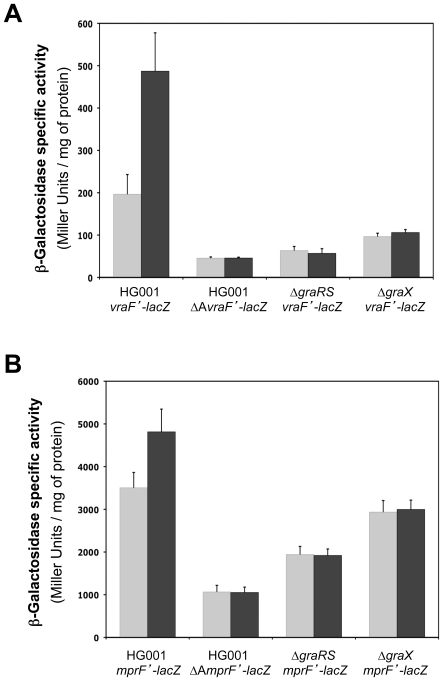
GraSR-dependent gene expression requires GraX, colistin and the consensus binding site. *vraFG* (A) and *mprF* (B) expression was followed using transcriptional *lacZ* fusions, with or without the upstream GraR operator sequence (*vraF*'-*lacZ*, *mprF*'-*lacZ* and ΔA*vraF*'-*lacZ*, ΔA*mprF*'-*lacZ*, respectively). The fusions were introduced in *S. aureus* strains HG001, ST1036 (Δ*graRS*) and ST1070 (Δ*graX*). Expression was measured during mid-exponential growth in TSB at 37°C (grey bars) or after treatment with 50 µg ml^−1^ colistin (black bars). β-Galactosidase assays were performed as described in Materials and Methods. Means and standard deviation values are presented from three independent experiments.

To determine the roles of GraX and this potential GraR-binding site in CAMP resistance, we constructed transcriptional *lacZ* fusions with the *vraFG* operon and *mprF* gene promoters, using the pSA14 vector, with or without the potential GraR operator sequence (*vraF*'-*lacZ* and ΔA*vraF*'-*lacZ* or *mprF*'-*lacZ* and ΔA*mprF*'-*lacZ*, respectively). The fusions were introduced into strains HG001, ST1036 (Δ*graRS*) and ST1070 (Δ*graX*) and β-galactosidase activity was measured during mid-exponential growth at 37°C in TSB with or without a 30 mn treatment with 50 µg ml^−1^ colistin (Fig. 4A). In the absence of GraX, GraSR or the potential GraR-binding site (strains ST1052 Δ*graX vraF*'-*lacZ*; ST1041 Δ*graRS vraF*'-*lacZ*; and ST1040 ΔA*vraF*'-*lacZ*), expression of *vraF*'-*lacZ* was strongly lowered, even in the presence of colistin (Fig. 4A). Comparable results were observed using the *mprF*'-*lacZ* and ΔA*mprF*'-*lacZ* fusions, although *mprF* clearly displays a higher basal level of expression in the absence of GraSR and GraX (Fig. 4B). We also note that there is a significant level of GraSR-dependent expression from both the *vraF* and *mprF* promoters in the absence of colistin, indicating that the GraSR system is at least partially active in the absence of inducer, or that it is responding to some other signal under these conditions.

As a control, we introduced a transcriptional fusion with the constitutively expressed promoter of the TU elongation factor *tufA* gene into *S*. *aureus* strain HG001, and no difference in *tufA'-lacZ* expression was observed after a 30 mn treatment with or without 200 µg ml^−1^ colistin (see Supplementary Material Fig. S1A). In order to rule out a potential colistin effect independent of its function as a CAMP, we measured β-galactosidase activities of the two fusions (*vraF*'*-lacZ* and *mprF*'-*lacZ*) in strain HG001 following 30 mn incubation in the presence or absence of 5 µg ml^−1^ indolicidin and found increased expression for both fusions upon addition of indolicidin (see Supplementary Material Fig. S1B).

**Figure 5:**
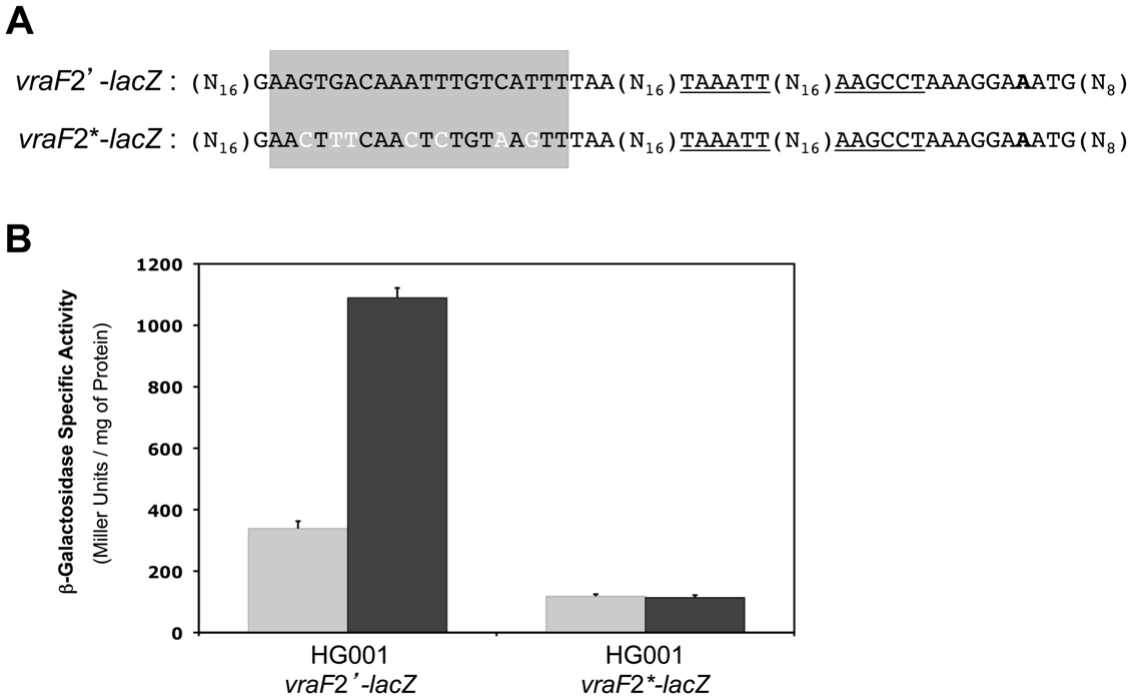
Point mutations in the GraR-binding site prevent *vraFG* expression and colistin induction. (A) Alignment of the DNA sequences used to construct the *vraF*2'-*lacZ* and *vraF*2*-*lacZ* fusions. Potential −35 and −10 sequences are underlined, the identified transcriptional start site is indicated in bold and the GraR-binding site is shaded. Point mutations introduced in the *vraF*2*-*lacZ* fusion are shown in white. (B) *vraF*2'-*lacZ* and *vraF*2*-*lacZ* fusion expression was measured in *S. aureus* HG001 (strains ST1168 and ST1169, respectively) during mid-exponential growth at 37°C in TSB (grey bars) or after treatment with 200 µg ml^−1^ colistin (black bars). β-Galactosidase assays were performed as described in Materials and Methods. Means and standard deviations values are presented from three independent experiments.

To further investigate the role of the GraR-binding site we have shown to be required for GraR-dependent regulation, we constructed two fusions of the same length between the *vraFG* operon promoter sequence and the *lacZ* gene in the pSA14 vector. The two fusions only extend nineteen base pairs upstream from the potential GraR binding site, and differ by the introduction of seven point mutations in the inverted repeat operator sequence by site-directed mutagenesis through PCR, effectively destroying the palindromic sequence (Fig. 5A). The native and mutated promoter fusions (*vraF*2*'*-*lacZ* and *vraF*2*'-*lacZ* respectively) were introduced into strain HG001 and β-galactosidase activity was measured during mid-exponential growth at 37°C in TSB with or without a 30 mn treatment with 200 µg ml^−1^ colistin (Fig. 5B). Expression of *vraF2*'-*lacZ* was induced by colistin (Fig. 5B, strain ST1168), whereas in strain ST1169 (HG001 *vraF*2*-*lacZ*) the mutations destroying the inverted repeat strongly diminished *vraF2*'-*lacZ* expression and induction by colistin was lost, leaving only a low basal level of expression similar to that measured in the absence of GraR or its binding site (Fig. 4A).

Taken together, these results show that GraSR, GraX and the inverted repeat sequence are all required for the expression of GraR-regulated genes and their induction in response to CAMPs, strongly suggesting that this sequence constitutes the GraR operator sequence. Although the exact function of GraX in this regulatory pathway remains to be elucidated, we show here that it acts as a cofactor of GraSR-dependent transcription activation.

### Genome-based prediction of the GraSR regulon

We then used the sequence of the identified likely GraR binding site to search for new potential GraSR-regulated genes in *S. aureus*. For this purpose, we used the restricted consensus 5′-ACAAAWKTGT-3′ to scan the *S. aureus* NCTC 8325 genome using the SearchPattern function of the ARTEMIS software [38]. To select GraSR-regulated candidate genes, we excluded inverted repeats lying more than 500 pb upstream from the annotated translational initiation site of each gene. We identified potential GraR-binding sites on either strand upstream from 29 genes or operons (Table 2). Among these, 13 had already been described in *S*. *aureus* and another 10 genes encode putative proteins whose potential function can be deduced from sequence similarities and are suggested to be involved in different cellular pathways, whereas the remaining genes are of unknown function (Table 2).

**Table 2:** Identification of potential new GraR regulon members by *in silico* genome scanning.

NCTC 8325^a^ (SAOUHSC_)	Gene or operon	DNA strand	Sequence^b^	Function^c^
			**→←**	**Antimicrobial resistance**
00867	*dlt* operon	+	**ACAAAATTGT…(N_186_) …TTG**	Teichoic acid D-alanylation
01359	*mprF*	+	**ACAAAAGTGT… (N_ 87_) …ATG**	Lysylphosphatidylglycerol synthetase
02751	*pnbA*	+	**ACAAAATTGT… (N_ 70_) …ATG**	Para-nitrobenzyl esterase
00667*	*vraFG* operon	+	**ACAAATTTGT… (N_150_) …GTG**	ABC transporter
				**Transport**
00167	*oppF*	−	**ACACATTTGT… (N_ 15_) …ATG**	Oligopeptide transporter ATP-binding protein
02815	*ycbE* (*B. amyloliquefaciens*)	−	**ACAATTTTGT… (N_ 85_) …ATG**	Probable glucarate transporter
00669		+	**ACAAATTTGT… (N_ 84_) …ATG**	Putative Pit family transporter
				**Cell envelope modification**
00992	*atlR (S.epidermidis)*	+	**ACAAATTTGT… (N_ 74_) …ATG**	Probable ATL autolysin transcription regulator
02571*	*ssaA*	+	**ACAAATTTGT…(N_264_)…ATG**	Amidase
00972	*tarM* operon	+	**ACAATTTTGT… (N_ 16_) …ATG**	Teichoic acid glycosylation
				**Oxidoreduction processes**
01818	*ald*	−	**ACAATTTTGT… (N_ 53_) …ATG**	Alanine dehydrogenase
01002*	*qoxABCD* operon	−	**ACAAATGTGT… (N_134_) …GTG**	Quinol oxidase AA3 subunit II
00035	*yrkE* (*B. pumilus*)	+	**ACAAATGTGT… (N_ 73_) …ATG**	Probable multidomain redox protein
01907	*ytbE (B. subtilis)*	−	**ACAAATTTGT… (N_279_) …ATG**	2,5-didehydrogluconate reductase
00882		−	**ACAAAATTGT… (N_250_) …ATG**	NADH dehydrogenase-like
				**Other functions**
01637	*comYC*	−	**ACAAATTTGT… (N_ 86_) …ATG**	Probable competence protein
02500	*rplE operon*	−	**ACAAATTTGT… (N_ 82_) …TTG**	50S ribosomal protein L5
02257	*sdrH*	−	**ACAAAATTGT… (N_505_) …ATG**	Atypical serine-aspartate rich (*sdr)* protein
00903	*spsB*	+	**ACAATTTTGT… (N_251_) …ATG**	Type-1 signal peptidase 1B
00776	*uvrB* operon	+	**ACAAATTTGT… (N_228_) …ATG**	Excinuclease
01819		+	**ACAAAATTGT… (N_ 67_) …ATG**	Universal stress protein UspA-like
02816		+	**ACAAAATTGT… (N_446_) …ATG**	Similar to alkaline phosphatase
00991	*ykrP* (*L. monocytogenes*)	−	**ACAAATTTGT… (N_ 58_) …ATG**	Probable acyltransferase
				**Unknown function**
00034		−	**ACACATTTGT… (N_ 43_) …ATG**	Conserved hypothetical protein
00146		−	**ACAAATTTGT… (N_104_) …ATG**	Probable transmembrane protein
00971		−	**ACAAAATTGT… (N_247_) …TTG**	Probable transmembrane protein
01242		+	**ACAAAATTGT… (N_223_) …ATG**	Conserved hypothetical protein
01851		−	**ACACATTTGT… (N_260_) …ATG**	Hypothetical protein
02320		−	**ACAAAATTGT… (N_129_) …ATG**	Hypothetical protein

a Gene names correspond to the annotation of the *S*. *aureus* NCTC 8325 genome sequence [69]. Only the first gene is indicated for operons.

b Positions of the inverted repeats of the potential GraR-binding sites are indicated for the given DNA strand with respect to the translation initiation codon.

c Known and putative functions for each regulon member based on genome annotations are indicated. Based on these predictions, the potential regulon members were divided in six categories (antimicrobial resistance, transport, cell envelope modification, oxidoreduction processes, other functions and unknown function).

*Indicates genes known or predicted to be controlled by the WalKR TCS [44], [46].

Interestingly, 15 genetic loci encode proteins that can be classified in major functional groups. The first includes known and putative antimicrobial resistance-associated proteins: MprF, DltABCD, VraFG, and a β-lactam antibiotic modifying enzyme named PnbA [39]. The second group corresponds to transport proteins: the oligopeptide ATP-binding transporter OppF, a putative glucaric acid transporter (SAOUHSC_02815) and two genes located directly downstream from the *vraFG* operon, encoding a putative inorganic phosphate transporter (SAOUHSC_00669 and SAOUHSC_00670) known as PitAB in *E. coli* [40]. In the third group, involved in cell envelope modification, we found the *tarM* operon involved in teichoic acid glycosylation [41] and genes encoding the cell wall amidase SsaA, a probable autolysin regulator (AtlR-like), and the SpsB signal peptidase. The fourth class of potentially GraR-regulated genes is linked to oxidoreduction processes, including the *qoxABCD* quinol oxidase operon, *ald,* an alanine dehydrogenase gene, and genes encoding the YrkE-like protein containing multi-redox domains and the YtbE-like protein probably involved in 2,5-didehydrogluconate reduction.

**Figure 6:**
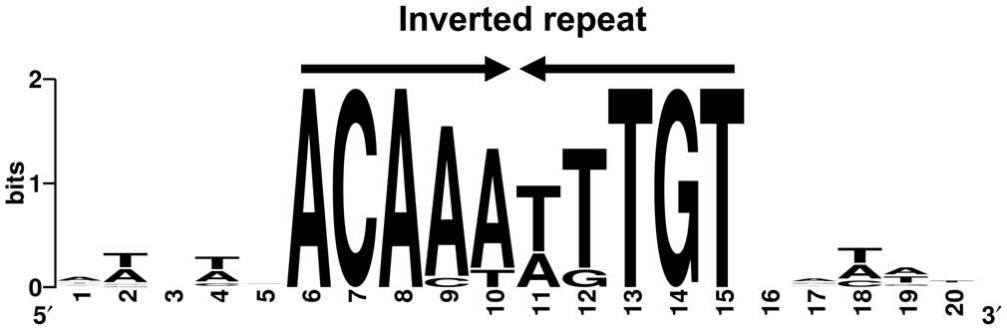
The GraR operator consensus is a perfect inverted repeat obtained by alignment of regulon gene upstream sequences. The consensus sequence for the GraR-binding site was generated using the WebLogo tool (http://weblogo.berkeley.edu/) by alignment of the upstream sequences of the 29 potential regulon genes identified by *in silico* analysis (Table 2).

Given the functional coherence of the identified loci, we investigated the relevance of this newly defined GraSR regulon by alignment of the 29 identified potential GraR-binding sites using the WebLogo website (http://weblogo.berkeley.edu/), generating a perfect 10 bp inverted repeat consensus sequence with a high degree of conservation, constituting the likely GraR operator: 5′ ACAAA TTTGT 3′ (Fig. 6).

### Expression profiling of the GraSR regulon

Having defined a consensus GraR operator sequence and several new potential members of the GraSR regulon, we wished to validate these predictions *in vivo*. Using the BaSysBio Sau T1 chip, a NimbleGen 385K feature tiling array designed to cover both strands of the entire *Staphylococcus aureus* NCTC 8325 genome (see Materials and Methods), we examined global expression changes between the parental HG001 strain and the Δ*graRS* mutant (ST1036), grown to mid-exponential phase in TSB with 50 µg ml^−1^ colistin. A total of 424 genes were found to be significantly differentially expressed in the Δ*graRS* mutant compared with the parental strain, with a ≥ 1.8-fold change in transcriptional levels and a P-value (Z-test) ≤3.5×10^−4^. Among these, 235 were positively controlled by GraSR and 189 were repressed (Table S1 and Table S2, respectively). Interestingly, among the positively controlled genes, the most highly regulated encode major virulence factors or regulators, while the remaining genes belonged to the principal categories uncovered by our *in silico* predictions (see Table 2; antimicrobial resistance, transport, cell envelope modification, oxidoreduction processes) as well as stress response genes, and multiple regulatory and metabolic pathways (acetate, purine and pyrimidine, pyridoxal, xanthine) (Tables S1 and S2). We chose to focus our attention on positively controlled genes involved in the classes uncovered by our *in silico* analysis, as well as virulence, regulation and stress response which are listed in Table 3.

**Table 3:** Expression profiling of the Δ*graRS* mutant.

Category/Gene name^a^		Function/similarity^b^	HG001/Δ*graRS* transcription ratio^c^	*P*-value
***Antimicrobial resistance***				
SAOUHSC_02611		HP similar to lysostaphin resistance protein A	3.7	<1.0E–16
SAOUHSC_01359	*mprF*	Phosphatidylglycerol lysyltransferase	2.8	<1.0E–16
SAOUHSC_00867		HP	2.7	<1.0E–16
SAOUHSC_00868	*dltX*	HP	2.0	1.04E–11
SAOUHSC_00869	*dltA*	D-alanine-D-alanyl carrier protein ligase	2.3	<1.0E–16
SAOUHSC_00871	*dltC*	D-alanine carrier protein	2.0	1.78E–11
SAOUHSC_00872	*dltD*	D-alanine-activating enzyme/transfer protein	2.1	1.38E–14
SAOUHSC_02866		HP similar to drug exporter of the RND superfamily	2.1	8.93E–14
SAOUHSC_02629		Putative EmrB/QacA family drug resistance transporter	1.8	1.16E–09
SAOUHSC_02630		HP similar to multidrug resistance protein A	1.9	3.42E–10
SAOUHSC_01866		HP similar to aminoglycoside resistance associated protein	1.8	5.96E–10
***Transport***				
SAOUHSC_02516		HP similar to major facilitator transporter permease	3.6	<1.0E–16
SAOUHSC_00060*		HP similar to Na-Pi cotransporter family protein	2.5	<1.0E–16
SAOUHSC_00136		HP similar to ABC transporter ATP-binding protein	2.5	<1.0E–16
SAOUHSC_00137		HP similar to sulfonate/nitrate/taurine transport system substrate-binding protein	2.0	1.41E–12
SAOUHSC_00138		HP similar to sulfonate/nitrate/taurine transport system permease	2.2	7.11E–15
SAOUHSC_00367		HP similar to proton/sodium-glutamate symporter	2.2	1.55E–15
SAOUHSC_02698		Putative amino acid ABC transporter permease	2.1	3.60E–14
SAOUHSC_02773		Putative aminobenzoyl-glutamate transporter	2.1	2.86E–13
SAOUHSC_00669*		HP similar to Pit family transporter	1.9	2.44E–10
SAOUHSC_02699		Putative amino acid ABC transporter ATP-binding protein	1.9	4.39E–12
SAOUHSC_02482	*cbiO*	Cobalt transporter ATP-binding subunit	1.8	5.57E–09
SAOUHSC_02733		HP similar to amino acid permesae	1.8	1.38E–09
***Cell envelope modification***				
SAOUHSC_02576*		HP CHAP domain-containing protein	18.2	<1.0E–16
SAOUHSC_02855		HP CHAP domain-containing protein	15.6	<1.0E–16
SAOUHSC_00427*	*sle1*	N-acetylmuramoyl-L-alanine amidase	14.9	<1.0E–16
SAOUHSC_02571*	*ssaA*	Secretory antigen SsaA precursor	8.0	<1.0E–16
SAOUHSC_00671*		HP CHAP domain-containing protein	5.8	<1.0E–16
SAOUHSC_02333*	*sceD*	Transglycosylase	4.9	<1.0E–16
SAOUHSC_00994*	*atlA*	Bifunctional autolysin	4.0	<1.0E–16
SAOUHSC_02887*	*isaA*	Immunodominant antigen A, transglycosylase	5.5	<1.0E–16
SAOUHSC_00256		HP CHAP domain-containing protein	2.3	<1.0E–16
SAOUHSC_02883*		HP CHAP domain-containing protein	2.2	3.11E–15
SAOUHSC_00974	*tarM*	wall teichoic acid glycosylation	1.8	1.17E–08
***Oxidoreduction processes***				
SAOUHSC_00999*	*qoxD*	Quinol oxidase, subunit IV	4.0	<1.0E–16
SAOUHSC_01000*	*qoxC*	Cytochrome c oxidase subunit III	3.5	<1.0E–16
SAOUHSC_01001*	*qoxB*	Quinol oxidase subunit I	4.3	<1.0E–16
SAOUHSC_01002*	*qoxA*	Quinol oxidase AA3 subunit II	6.9	<1.0E–16
SAOUHSC_01103	*sdhC*	Succinate dehydrogenase cytochrome b-558 subunit	1.8	2.66E–15
SAOUHSC_01104	*sdhA*	Succinate dehydrogenase flavoprotein subunit	1.9	3.73E–11
SAOUHSC_01105	*sdhB*	Succinate dehydrogenase iron-sulfur subunit	1.8	1.99E–09
***Stress***				
SAOUHSC_00634	*mntC*	Putative ABC transporter substrate-binding protein	3.9	<1.0E–16
SAOUHSC_00636	*mntB*	Putative iron (chelated) ABC transporter permease	4.3	<1.0E-16
SAOUHSC_00637	*mntA*	Putative manganese/iron ABC transporter ATP-binding protein	2.5	<1.0E–16
SAOUHSC_03045	*cspB*	Cold shock protein CspB	2.3	<1.0E–16
SAOUHSC_01730	*csbD*	Sigma-B mediated bacterial general stress response protein	2.1	<1.0E–16
SAOUHSC_00819	*cspC*	Cold shock protein C	1.9	1.18E–13
SAOUHSC_01403	*cspA*	Cold shock protein	1.9	1.13E–10
***Regulation***				
SAOUHSC_02261	*agrB*	Accessory gene regulator protein B	21.6	<1.0E–16
SAOUHSC_02262	*agrD*	Accessory gene regulator protein D	21.0	<1.0E–16
SAOUHSC_02264	*agrC*	Accessory gene regulator protein C	9.3	<1.0E–16
SAOUHSC_02265	*agrA*	Accessory gene regulator protein A	4.2	<1.0E–16
SAOUHSC_02566	*sarR*	Staphylococcal accessory regulator R	3.4	<1.0E–16
SAOUHSC_00674	*sarX*	Staphylococcal accessory regulator protein X	3.1	<1.0E–16
SAOUHSC_00070	*sarS*	Staphylococcal accessory regulator A-like	2.7	<1.0E–16
SAOUHSC_00230	*lytS*	Two-component sensor histidine kinase	2.0	3.21E–12
SAOUHSC_00231	*lytR*	Two-component response regulator	2.0	5.40E–12
SAOUHSC_01891	*arsR*	Arsenical resistance operon repressor	1.8	5.60E–10
SAOUHSC_02388	*czrA*	Metal-dependent transcriptional regulator	1.8	7.31E–09
***Virulence factors***				
SAOUHSC_01135		HP SLUSH-like haemolytic protein	44.6	<1.0E–16
SAOUHSC_01136		HP SLUSH-like haemolytic protein	98.4	<1.0E–16
SAOUHSC_02260	*hld*	Delta-haemolysin	80.1	<1.0E–16
SAOUHSC_01110		Putative fibrinogen-binding protein	8.5	<1.0E–16
SAOUHSC_00300	*geh*	Lipase precursor	4.7	<1.0E–16
SAOUHSC_01953	*epiA*	Lantibiotic epidermin precursor EpiA	3.2	<1.0E–16
SAOUHSC_02163	*hlb*	Beta-haemolysin	2.8	5.10E–11
SAOUHSC_02167		HP Similar to complement inhibitor protein SCIN	2.8	<1.0E–16
SAOUHSC_01121	*hla*	Alpha-haemolysin	2.5	<1.0E–16
SAOUHSC_02169	*chp*	Chemotaxis-inhibiting protein CHIPS	2.4	<1.0E–16
SAOUHSC_02963	*clfB*	Clumping factor ClfB	2.3	2.22E–16
SAOUHSC_01114	*efb*	Fibrinogen-binding protein	1.9	4.59E–10
SAOUHSC_02257	*sdrH*	Atypical serine-aspartate rich (*sdr*) protein	1.9	2.84E–10
SAOUHSC_02706	*sbi*	Immunoglobulin G-binding protein	1.8	2.77E–09

a Gene names correspond to the annotation of the *S*. *aureus* NCTC 8325 genome sequence [69].

b HP: hypothetical protein.

c Fold-change of selected positively regulated genes determined as the ratio of the signal values between strain HG001 and the Δ*graRS* mutant. * Indicates genes known or predicted to be controlled by the WalKR TCS [44], [46].

The most strongly regulated genes encode haemolysins, the AgrBDCA peptide quorum-sensing signal transduction pathway, members of the Sar family of virulence regulators, several host interaction proteins and virulence factors, (fibrinogen binding protein, ClfB, CHIPS, haemolysins, Sbi, SdrH), autolysins, as well as quinol oxidases (Table 3). This is the first report linking GraSR and the AgrCA major *S*. *aureus* virulence regulatory system.

Among the regulatory genes, we note those encoding the LytSR TCS, involved in autolysis and biofilm formation [42], [43]. Most of the genes involved in cell envelope modification encode autolysins, including the AtlA major bifunctional autolysin, the SceD and IsaA transglycosylases, as well as seven genes encoding potential amidases with CHAP domains (Cysteine, Histidine-dependent Amidohydrolases/Peptidases), such as Sle1 or SsaA (Table 3). Interestingly, eight of the GraSR-dependent autolysin genes also belong to the WalKR regulon [44], [45] (indicated by an asterisk in Table 3) suggesting a significant regulatory overlap between the two cell envelope signal transduction pathways. Indeed, thirteen other members of the GraSR regulon have also been predicted as belonging to the WalKR regulon as they are preceded by a consensus binding site for the WalR response regulator [44], [46], such as the *qoxABCD* and SAOUHSC_00669-SAOUHSC_00670 operons (indicated by asterisks in Tables 2 & 3, S1 & S2).

**Figure 7:**
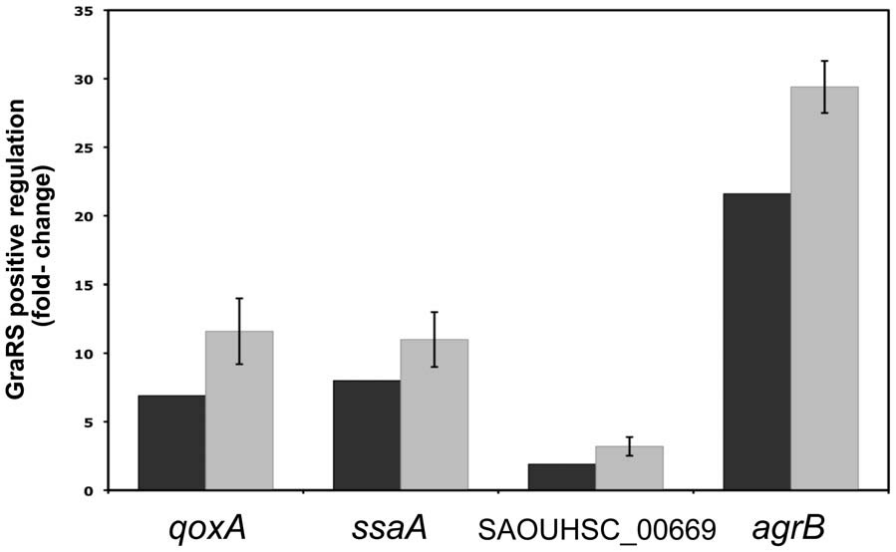
Correlation between microarray and qRT-PCR experiments for expression of GraSR-dependent genes. The expression levels of *qoxA*, *ssaA*, SAOUHSC_00669 and *agrB* genes were analyzed by qRT-PCR in the HG001 and ST1036 (Δ*graRS*) strains. RNA samples were prepared from cultures during mid exponential growth after treatment with 50 µg ml^−1^ colistin. Comparative analysis (fold-change) of transcriptome analysis (black bars) and qRT-PCR experiments (grey bars) are shown. Means and standard deviation values for the qRT-PCR data are presented from at least three independent experiments.

In order to validate our microarray data, we chose several relevant genes (*qoxA*, *ssaA*, SAOUHSC_00669 and *agrB*) and compared their relative expression levels in the parental HG001 strain and the Δ*graRS* mutant by qRT-PCR. As shown in Fig. 7, we confirmed by qRT-PCR that all of these genes are positively controlled by GraSR, with factors higher than those seen in the transcriptome analysis, ranging from approximately 3- to 29-fold. Results obtained using the two methods showed a linear correlation (Fig. S2; see Supplementary Material).

### GraXSR are involved in *S. aureus* resistance to oxidative stress

**Figure 8:**
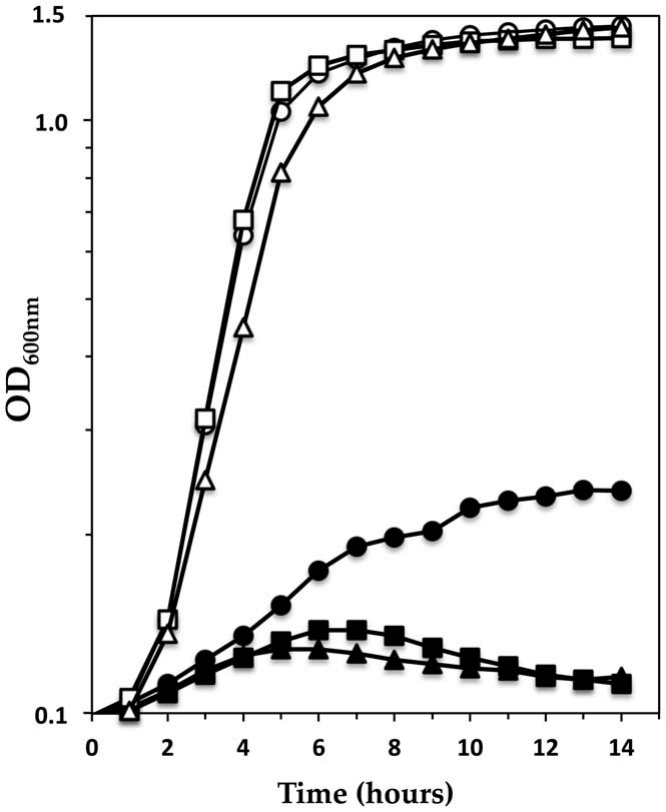
GraXSR are involved in oxidative stress resistance. The effect of 40 mM paraquat was analyzed on HG001 (•, ○), ST1036 (▪, □; Δ*graRS*), ST1070 (▴, ▵; Δ*graX*) strains grown in TSB at 37°C, and diluted to a final OD _600 nm_ of 0.025. Growth was followed at 600 nm using a microtiter plate reader in the presence (closed symbols) or absence (open symbols) of 40 mM paraquat (methylviologen). A representative curve of three independent experiments is shown for each strain.

Since GraSR control the expression of genes that appear to involved in oxidoreduction processes, we compared the sensitivity to oxidative stress of the parental HG001 strain with that of the ST1036 (Δ*graRS*) and ST1070 (Δ*graX*) mutants. Cells were grown in TSB in the presence or absence of 40 mM paraquat (methylviologen). No significant difference in growth between the strains was observed in the absence of paraquat (Fig. 8, open symbols). However, as shown in Fig. 8 (closed symbols), the Δ*graX* and Δ*graRS* mutants were much more strongly affected by the presence of paraquat than the HG001 parental strain. Moreover, similar results were obtained in the presence of H_2_O_2_ for the Δ*graRS* mutant (data not shown). These results reveal a novel function for the GraSR system in resistance of *S*. *aureus* to oxidative stress.

### The GraSR system is required for growth of *S. aureus* at high temperature

**Figure 9:**
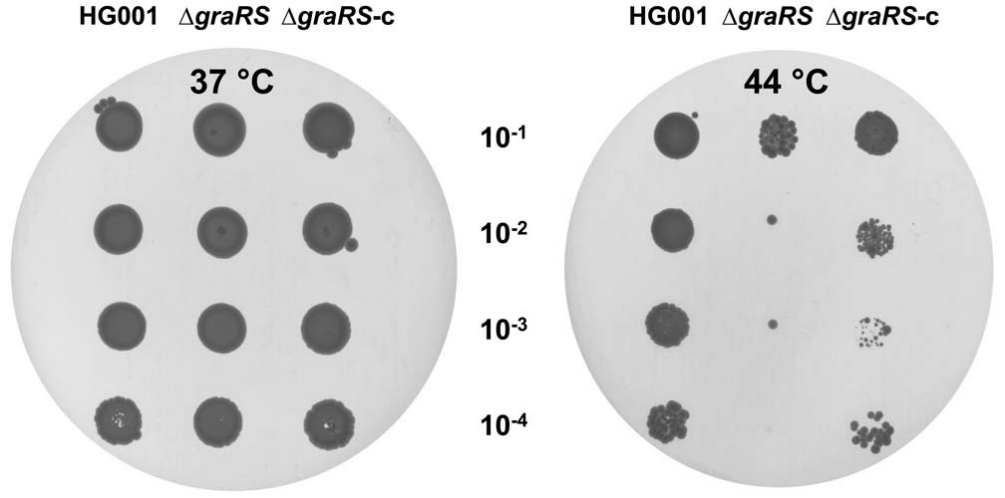
GraSR are required for growth of *Staphylococcus aureus* at high temperature. The effect of high temperature was tested on growth of *S. aureus* strains ST1120 (HG001 pMK4-Pprot), ST1117 (Δ*graRS* pMK4-Pprot) and the complemented Δ*graRS* mutant, Δ*graRS*-c (strain ST1116; Δ*graRS* pMK4-Pprot*-graR*). Strains were grown at 37°C in TSB and diluted to an OD _600 nm_ of 0.2. Serial dilutions were then carried out and 10 µl of each dilution was spotted on TSA plates, and incubated at 37°C or 44°C for 48 h.

The GraSR system is involved in cell envelope modifications through regulation of *mprF*, the *dlt* operon and autolysin genes, and also controls the expression of stress response genes (Tables 3, S1 & S2). We therefore tested the ability of the Δ*graRS* mutant to grow at high temperatures using a plate spotting assay. Strains ST1120 (HG001 pMK4-Pprot), ST1117 (Δ*graRS* pMK4-Pprot) and the complemented Δ*graRS* mutant, Δ*graRS*-c (Strain ST1116 Δ*graRS* pMK4-Pprot*-graR*) were grown in TSB at 37°C and diluted to an OD _600 nm_ of 0.2. Serial dilutions were then carried out, spotted on TSA plates and incubated at 37°C or 44°C for 48 h. As shown in Fig. 9, growth of the Δ*graRS* mutant was strongly impaired at 44°C as compared to the parental strain, whereas no differences were observed between the two strains at 37°C. Resistance to high temperatures was almost completely restored in the complemented Δ*graRS*-c strain carrying the *graR* gene on a multicopy plasmid, indicating that this phenotype can be compensated by overproducing the response regulator alone. These results demonstrate an important role for the GraSR system in growth of *S*. *aureus* at high temperatures.

## Discussion

Cationic antimicrobial peptides (CAMPs) are a major component of host innate immune defense systems, produced by all living organisms, and have emerged as promising therapeutic antimicrobial agents [47], [48]. Part of the success of some major human pathogens such as *Staphylococcus aureus* can be attributed to efficient CAMP resistance. One such mechanism involves incorporation of positively charged residues into the envelope, effectively increasing electrostatic repulsion of CAMPs from the cell surface. In *S*. *aureus*, this is accomplished through D-alanylation of teichoic acids, mediated by the DltABCD enzymes, as well as by MprF-dependent lysylination of phosphatidylglycerol [10]. Expression of *mprF* and the *dlt* operon is induced by the presence of CAMPs and specifically controlled by the GraSR TCS, which has attracted growing interest in recent years [16], [17], [18], [19]. GraSR also control expression of the *vraFG* operon, located directly downstream of the *graXRS* genes and encoding an ABC transporter also playing a role in CAMP resistance [16], [17], [18], [19].

In this study we wished to further define the GraSR regulon and its function. We determined that unlike other TCSs such as VraSR or AgrCA [13], [30], GraSR do not autoregulate their own synthesis. We demonstrated that *graX*, cotranscribed with *graRS,* specific to *S. aureus* and also involved in CAMP resistance, encodes an essential regulatory cofactor of the GraSR signaling pathway, effectively constituting a three-component system.

Noting that an imperfect palindromic sequence upstream from the *vraFG* operon had been suggested as a potential regulatory target in a multi-genome analysis of low G+C% Gram-positive bacteria [37], we found this inverted repeat as being highly conserved upstream from two other well-studied GraSR target genes/operons, *mprF* and *dltXABCD*. Extending our analysis through detailed genome scanning of the *S*. *aureus* NCTC 8325 genome, we were able to derive a highly conserved ten base pair perfect palindromic sequence (5′ ACAAA TTTGT 3′) upstream from 29 potential GraSR regulon members (Table 2). By a genetic approach combining deletions and point mutations, we were able to conclusively demonstrate that this sequence is essential for transcriptional regulation by GraR and induction in response to CAMPs, indicating it is the likely GraR operator binding site. Despite multiple attempts, we were unable to purify the GraR response regulator in an active form in order to show DNA-binding *in vitro*. However, our proposed GraR operator binding site is similar to that suggested for the closely related VirR response regulator of *Listeria monocytogenes* which was shown to bind to DNA [35]. The two proteins share 46% overall amino acid sequence identity, rising to 73% for the winged helix-turn-helix domain, with 9 out of 11 identical residues in the DNA recognition helix, indicating that they must bind to similar DNA sequences.

Of the 29 potential GraR regulon members we identified with this binding site present in their upstream regions, we showed that 13 of these are indeed controlled by the GraSR system *in vivo* under our conditions. Of these, nine are positively regulated (*dltXABCD*, *mprF*, *vraFG*, *tarM*, *sdrH*, *ssaA*, SAOUHSC_00669, *qoxABCD*, *rplE*) and four were found to be repressed (SAOUHSC_00146, SAOUHSC_00991, SAOUHSC_00882, SAOUHSC_02816). This suggests that for the remaining 16 predicted target genes, either the potential GraR binding site is not appropriately located with respect to the promoter in order to allow transcriptional activation/repression, or that additional genetic control mechanisms exist for these genes, preventing their expression under our specific experimental conditions.

During a phenotypic analysis, we observed that the Δ*graRS* mutant displayed increased sensitivity to oxidative stress. This may in part be due to positive control by GraSR of the *mntABC* manganese transporter genes (Table 3), which have been shown to play a role in *S*. *aureus* resistance to superoxide radicals [49]. Furthermore, these data suggest that the GraSR system may also respond to other signals in *S. aureus*, and not only to the presence of CAMPs. The combined sensitivity of the Δ*graRS* mutant to oxidative stress and antimicrobial peptides could explain the important role of this system in staphylococcal survival in human neutrophils [23], [24].

Virulence gene expression in *S. aureus* involves a complex regulatory network, with at least four two-component systems (AgrCA, ArlSR, SaeSR and SrrAB) and several accessory transcription factors (SarA, SarS, SarT, SarR, and Rot) [13]. In this study, our transcriptome analysis allowed us to unveil previously unsuspected connections between the GraSR TCS and the AgrCA signal transduction network. Indeed, expression of the *agrBDCA*, *hla*, *hlb*, *hld*, *sarR*, *sarS*, and *sarX* genes are all strongly lowered in the Δ*graRS* mutant (Table 3), as well as those of several other genes encoding virulence factors (CHIPs, ClfB, lipase, Sbi, etc.). Part of this effect may be due to Rsr which represses expression of *sarR*, *agr* and *hla* [50]. Indeed, expression of *rsr* is itself repressed by GraSR, increasing approximately 2.6-fold in the Δ*graRS* mutant (Table S2). This is the first report linking the GraSR and AgrCA TCSs, which could in part explain the numerous results implicating the GraSR system in *S*. *aureus* virulence using several experimental models [18], [23]–[26]. It is likely this connection could not be detected in a previous GraSR transcriptome analysis as it was carried out using strain SA113, an *agr* mutant, in the absence of CAMPs as an inducer [16], [27]. However, comparing the two sets of transcriptome data obtained during exponential growth revealed similar numbers of GraSR-regulated genes using the same cutoff values, although significant differences in the genes controlled were observed. Indeed, only 63 genes were common to the two experiments, and 19 differentially regulated in each condition, which may be attributed to differences in the genetic backgrounds of the two strains (HG001 and SA113) or to a different behaviour of the GraSR system under basal growth conditions or upon induction in the presence of CAMPs.

In addition to AgrCA, GraSR also appear to interact with the WalKR TCS, involved in cell wall metabolism and autolysis [45], [51], providing increasing evidence for TCS signal transduction networking in *S*. *aureus*, as described for the Gram-positive model bacterium *B. subtilis* [52]. Regulatory overlap with the WalKR regulon is particularly extensive, with at least 21 WalR regulon genes also controlled by GraSR, suggesting a significant level of interaction between the two cell envelope signal transduction pathways. These include eight autolysin genes (*atlA*, *sceD*, *isaA*, *ssaA*, *sle1*, SAOUHSC_00671, SAOUHSC_02576, SAOUHSC_02883) that have all been shown to be transcriptionally controlled by WalKR [45] (Delaune *et al*., in preparation). Thirteen other members of the GraSR regulon have also been predicted as being controlled by WalKR as they are preceded by a consensus binding site for the WalR response regulator, including the *qoxABCD* quinol oxidase biosynthesis operon, the SAOUHSC_00669-SAOUHSC_00670 operon, *prs*, encoding a putative ribose-phosphate pyrophosphokinase, the *manA* mannose-6 phosphate isomerase gene, the SAOUHSC_00738 and *vraFG* ABC transporter genes as well as a gene of unknown function, SAOUHSC_ 00060 (Tables 3, S1 & S2) [44], [46]. This is reminiscent of genes under multiple regulatory controls, such as the *B*. *subtilis degQ* gene, which is preceded by tandemly arranged binding sites for both the DegU and ComA response regulators [53], and it will be interesting to determine the respective contributions of WalKR and GraSR to expression of their co-regulated genes.

During this investigation, we also showed that GraSR are required for growth of *S. aureus* at high temperatures. This may be linked to their role in modification of wall teichoic acids, which are known to be required for growth under these conditions [54]. This is the first report revealing a function for GraX as a regulatory cofactor of the GraSR TCS, and showing a role for this system in staphylococcal high temperature and oxidative stress survival. We have shown that the GraSR system controls genes involved in stress response, cell wall metabolism and pathogenesis control pathways in addition to its primary role in CAMP resistance, significantly enhancing its importance as a major signal transduction pathway in *S*. *aureus*.

## Materials and Methods

### Bacterial strains and growth procedures

Bacterial strains and plasmids are listed in Table 4. *Escherichia coli* K12 strain DH5α™(Invitrogen Life Technologies) was used for cloning experiments. Plasmid constructs were first passaged through the restriction modification deficient *S. aureus* strain RN4220 [55] before introduction into *S. aureus* strain HG001, a *rsbU* ^+^ variant of strain NCTC 8325 [27] and its derivatives. HG001 is a genetically tractable, clinically relevant non mutagenized strain and was used for all genetic studies. *E. coli* strains were grown in LB medium with ampicillin (100 µg ml^−1^) when required. *S. aureus* was grown in Trypticase Soy Broth (TSB; Difco) with shaking (180 rpm) at 37°C ; for plasmid selection, chloramphenicol (10 µg ml^−1^) or erythromycin (2 µg ml^−1^) were added as required. *E*. *coli* and *S*. *aureus* strains were transformed by electroporation using standard protocols [56] and transformants were selected on LB or Trypticase Soy Agar (TSA; Difco) plates, respectively, with the appropriate antibiotics. Colistin sulfate, nisin and indolicidin (Sigma-Aldrich) were used as CAMPs when required.

**Table 4:** Bacterial strains and plasmids used in this study.

Strain or plasmid	Relevant genotype or description	Source or construction^a^
**Strains**		
*Escherichia coli*		
DH5α™	F^-^ [Φ80 (d*lac*ZΔM15) Δ (*lac*ZYA-*arg*F) U169 *rec*A1 *end*A1 *hsd*R17 (r_K_-, m_K_+) *pho*A *sup*E44 λ– *thi*-1 *gyr*A96 *rel*A1	Invitrogen Life Technologies
*Staphylococcus aureus*		
RN4220	Restriction deficient transformation recipient	[55]
HG001	NCTC 8325 *rsbU* ^+^	[27]
ST1036	Δ*graRS*	pMAD*graRS*→ HG001
ST1039	*vraF*'-*lacZ,* Cm^r^	pSA14*vraF* → HG001
ST1040	ΔA*vraF*'-*lacZ,* Cm^r^	pSA14ΔA*vraF* → HG001
ST1041	Δ*graRS vraF*'-*lacZ,* Cm^r^	pSA14*vraF* → ST1036
ST1052	Δ*graX vraF*'-*lacZ,* Cm^r^	pSA14*vraF* → ST1070
ST1070	Δ*graX*	pMAD*graX*→ HG001
ST1074	*mprF*'-*lacZ,* Cm^r^	pSA14*mprF* → HG001
ST1075	Δ*graRS mprF*'-*lacZ,* Cm^r^	pSA14*mprF* → ST1036
ST1082	*graX*'-*lacZ,* Cm^r^	pSA14*graX* → HG001
ST1105	Δ*graX mprF*'-*lacZ,* Cm^r^	pSA14*mprF* → ST1070
ST1111	ΔA*mprF*'-*lacZ,* Cm^r^	pSA14ΔA*mprF* → HG001
ST1112	Δ*graRS graX*'-*lacZ,* Cm^r^	pSA14*graX* → ST1036
ST1116	Δ*graRS* pMK4-Pprot*graR,* Cm^r^	pMK4-Pprot*graR* → ST1036
ST1117	Δ*graRS* pMK4-Pprot, Cm^r^	pMK4-Pprot → ST1036
ST1120	pMK4-Pprot, Cm^r^	pMK4-Pprot → HG001
ST1168	*vraF*2'-*lacZ,* Cm^r^	pSA14*vraF*2 → HG001
ST1169	*vraF*2*-*lacZ,* Cm^r^	pSA14*vraF*2* → HG001
ST1176	Δ*graX graX*'-*lacZ,* Cm^r^	pSA14*graX* → ST1070
ST1199	*PtufA*'-*lacZ,* Cm^r^	pSA14*tufA*→ HG001
**Plasmids**		
pMAD	pE194 derivative with a thermosensitive origin of replication for deletion/replacement of genes in Gram-positive bacteria	[57]
pMAD*graRS*	pMAD derivative allowing deletion of the *graRS* genes	This study
pMAD*graX*	pMAD derivative allowing deletion of the *graX* gene	This study
pSA14	pMK4 derivative carrying the promoterless *E*. *coli lacZ* gene for constructing transcriptional fusions	This study
pSA14*vraF*1	pSA14 derivative carrying the intergenic region between *graXRS* and *vraFG*	This study
pSA14ΔA*vraF*1	pSA14 derivative carrying the truncated intergenic region between *graXRS* and *vraFG*	This study
pSA14*mprF*	pSA14 derivative carrying the *mprF* promoter region	This study
pSA14ΔA*mprF*	pSA14 derivative carrying the *mprF* truncated promoter	This study
pSA14*graX*	pSA14 derivative carrying the *graX* promoter region	This study
pSA14*vraF*2	pSA14 derivative carrying the *vraFG* promoter region	This study
pSA14*vraF*2*	pSA14 derivative carrying the truncated *vraFG* promoter region	This study
pSA14*tufA*	pSA14 derivative carrying the *tufA* promoter region	[63]
pMK4Pprot	pMK4 derivative carrying a constitutive Gram-positive promoter for gene complementation	[58]
pMK4-Pprot*graR*	pMK4-Pprot derivative carrying *graR*	This study

a Arrows indicate plasmid introduction by electroporation.

### DNA manipulations

Oligonucleotides used in this study were synthesized by Sigma-Proligo and are listed in Table 5. *S*. *aureus* HG001 chromosomal DNA was isolated using the MasterPure™Gram-positive DNA purification Kit (Epicentre Biotechnologies). Plasmids were isolated using a QIAprep Spin Miniprep kit (Qiagen) and PCR fragments were purified using the Qiaquick PCR purification kit (Qiagen). T4 DNA ligase and restriction enzymes (New England Biolabs), PCR reagents and High-Fidelity *Pwo* thermostable DNA Polymerase (Roche) were used according to the manufacturers' recommendations. Nucleotide sequencing of plasmid constructs was carried out by Genome Express-Cogenics or GATC Biotech.

**Table 5:** Oligonucleotides used in this study*.

Name	Sequence	Description
MF9	5′-GTC*GTCGAC*GAGCAGCGGCTATCAATCAA-3′	*graRS* upstream region, deletion mutant
MF10	5′-CTC*CTCGAG*TATTTGCATCCATATCACCC-3′	
MF11	5′-CTC*CTCGAG*TTGAACGCATGTCGGAAGT-3′	*graRS* downstream region, deletion mutant
MF12	5′-AGA*AGATCT*GCACCTGTTGGTTCGTCAGC-3′	
MF35	5′-GAA*GAATTC*GTCATCATAATATTTACCATCG-3′	*graX* upstream region, deletion mutant
MF36	5′-GGT*GGTCTC*TCACATCTAAAATACTCCTT-3′	
MF37	5′-GGT*GGTCTC*ATGTGATATTGGGTGATATG-3′	*graX* downstream region, deletion mutant
MF38	5′-CCA*CCATGG*TTAACGTATTATCACTAACA-3′	
MF62	5′-CGCTAACATTGAAATGAAATTTTCTACATC-3′	*graXRS* promoter region, primer extension
MF63	5′-ACCAATATATCCTGTTCCACCTGCTAATAA-3′	
MF66	5′-ATGAAATTGTTGAACGCATGTCGGAAGT-3′	*vraFG* promoter region, primer extension
MF67	5′-TCGCAACACTTCTTGTGCCATTTTTTTAGT-3′	
MF69	5′-AGATTTCATATTGCACCTCTTAAAGTTC-3′	*dltXABCD* promoter region, primer extension
MF70	5′-GCGGTTATGACAAATCAGGTACCACATACT-3′	
MF74	5′-CTG*CTGCAG*GGGACAACTGTCAGATTGATT-3′	*graXRS* and *vraFG* intergenic region (*vraF1*), *lacZ* fusions
MF75	5′-GGA*GGATCC*TTAACTTCATTTCCTTTAGGC-3′	
MF77	5′-GTATAGATAACCATATTGTTCTGTTTGAGA-3′	*mprF* promoter region, primer extension
MF78	5′-GATAACTCCCGATACAATGTGATTGCTACA-3′	
MF88	5′-CTG*CTGCAG*TTTAAACATGCGTTTTGTT-3′	*graXRS* and *vraFG* truncated intergenic region (*vraF*1), *lacZ* fusions
MF89	5′-CTG*CTGCAG*TATAGATAACCATATTGTTC-3′	*mprF* promoter region, *lacZ* fusions
MF90	5′-GGA*GGATCC*TGATTCATTTTTTCACATCA-3′	
MF91	5′-CTG*CTGCAG*TATAAATCAAAGGTAAATG-3′	*mprF* truncated promoter region, *lacZ* fusions
MF95	5′-CTG*CTGCAG*ATTCGTCGTATATGTTCGCT-3′	*graXRS* promoter region, *lacZ* fusions
MF96	5′-GGA*GGATCC*AATATATCCTGTTCCACCTGCT-3′	
MF97	5′-CTG*CTGCAG*TCGTTCGGTTATGCAA-3′	*tufA* promoter region, complementation
MF98	5′-GGA*GGATCC*ACTCTCTCATGATAGTTTCT-3′	
MF118	5′-GGA*GGATCC*GGAGGTGATATGGATGCAAATAC-3′	*graR* coding sequence, complementation
MF119	5′-CTG*CTGCAG*TTATTCATGAGCCATATATCCTT-3′	
OSA161	5′-TACCTTACCAACTAGCTAATGCAGCG-3′	16S intragenic region, qRT-PCR
OSA162	5′-ACGTGGATAACCTACCTATAAGACTGGGAT-3′	
OAH131	5′-TGTTGACTGCAGGTCGGAACTTTCAACTCTGTAAGTTAAACATGCG-3′	*vraFG* promoter region (*vraF*2), *lacZ* fusions
OAH132	5′-TGTTGA*CTGCAG*GTCGGAAGTGACAAATTTGTC-3′	*vraFG* mutated promoter region (*vraF*2*), *lacZ* fusions

*Added restriction site sequences are indicated in italics.

### Plasmid and mutant construction

The thermosensitive shuttle vector pMAD was used for introducing markerless gene deletions [57]. Mutant strains of *S. aureus* HG001 used in this study were obtained by gene deletions, removing the entire coding sequence without the introduction of an antibiotic resistance gene. In a first step, two DNA fragments, of approximately 600 bp, corresponding to the chromosomal DNA regions located directly upstream and downstream from the gene(s) of interest, were generated by PCR, digested with *Xho*I or *Bsa*I, and ligated using T4 DNA ligase. The ligation product was reamplified using the external primers and purified before cloning into the temperature-sensitive shuttle vector pMAD between the *Eco*RI/*Nco*I or *Sal*I/*Bgl*II restriction sites. Nucleotide sequences of the constructs were confirmed by DNA sequencing and the resulting plasmids passaged through *S. aureus* strain RN4220 and introduced into *S. aureus* HG001. Integration and excision of the pMAD derivatives and deletion of the chromosomal region of interest was carried out as previously described [57]. Gene deletions in mutant strains were systematically verified by PCR and qRT-PCR.

Plasmid pMK4Pprot, a derivative of vector pMK4 carrying a constitutively expressed Gram-positive promoter sequence [58] was used for gene complementation experiments. Complementation of the ST1036 (Δ*graRS*) strain was carried out using a DNA fragment corresponding to the *graR* gene coding sequence, amplified with oligonucleotides MF118-MF119, generating *Bam*HI/*Pst*I restriction sites at the extremities, and cloned in the replicative plasmid pMK4-Pprot.

Plasmid pSA14 was used to measure the expression of *S. aureus* genes by constructing transcriptional fusions between gene promoter regions and the *E. coli lacZ* reporter gene. The pSA14 plasmid was constructed by cloning a 3.2 kb *Eco*RI-*Pst*I DNA fragment from plasmid pHT304-18Z [59] between the corresponding restriction sites of the pMK4 shuttle vector [60]. The insert carried the promoterless *E. coli lacZ* gene fused to the *B. subtilis spoVG* ribosome binding site [61], [62]. In the absence of an upstream promoter, the pSA14 vector displays no detectable β-galactosidase activity, making it a highly useful transcriptional *lacZ* fusion reporter tool for *in vivo* expression analysis. For constructing transcriptional *lacZ* fusions, promoter regions of the *mprF* gene and the *graXRS* and *vraFG* operons, and truncated promoter regions of *mprF* gene and the *vraFG* operon were amplified by PCR using oligonucleotides introducing *Bam*HI/*Pst*I restriction sites, except for the *vraF*2*-*lacZ* fusion, constructed using a forward oligonucleotide containing seven mismatches generating point mutations (see Table 5). The corresponding DNA fragments were then cloned between the corresponding restriction sites of the pSA14 vector, yielding plasmids listed in Table 4. The pSA*tufA* plasmid [63] carrying the strong constitutive promoter of the *tufA* gene was used as a control.

### MIC determinations

MIC determinations were performed in a 96-well microtiter plate with a 100 µl culture volume. Bacterial cultures were grown for eight hours in TSB at 37°C, diluted to an OD _600 nm_ = 0.05 and used to inoculate wells containing TSB with standard two-fold increments of colistin concentration (v/v). Plates were incubated for 12 h with vigorous shaking at 37°C in a Synergy 2 thermoregulated spectrophotometer plate reader using the Gen5™Microplate Software (BioTek Instruments Inc., Winooski, VT). All experiments were performed in triplicate.

### Extraction of total RNA

Cells were grown until OD _600 nm_  = 1, and colistin was added to the medium at 50 µg ml^−1^ or 200 µg ml^−1^ when required. Growth was pursued during 30 min and cells were harvested by centrifuging 30 ml culture samples (4 min; 5,400 x *g*) and immediately frozen at −80°C. RNA extractions were then performed as previously described [64], followed by a DNase I treatment with the TURBO DNA-free reagent (Ambion, Austin, TX) in order to eliminate residual contaminating genomic DNA.

### Primer extensions

Primer extensions were performed as previously described [65] using 30 µg of RNA, 2 pmol of oligonucleotide (previously radiolabeled with [γ-^32^P] ATP using T4 polynucleotide kinase, New England Biolabs), and 200 U of Superscript II reverse transcriptase (Invitrogen). Oligonucleotides were chosen so as to hybridize downstream from the translation initiation codon (see Table 5). The corresponding DNA sequencing reactions were carried out with the same oligonucleotides and PCR-amplified DNA fragments carrying the respective promoter regions, using the Sequenase PCR product sequencing kit (USB, Cleveland, OH).

### β-Galactosidase assays

Cells were grown until OD _600 nm_  = 1, colistin was added to the medium at 50 µg ml^−1^ or 200 µg ml^−1^ when required and growth was pursued for 30 min. *S*. *aureus* strains carrying the different *lacZ* fusions were then harvested by centrifuging 2 ml culture samples (2 min; 20,800 x *g*). Cells were resuspended in 500 µl of Z buffer [66] with 0.5 mg ml^−1^ DNase, 5 mM DTT and 0.1 mg ml^−1^ lysostaphin added extemporaneously, and lysed by incubation at 37°C for 30 min. Cell debris were eliminated by centrifugation (2 min; 20,800 x *g*) and the supernatant was either used directly for assays or stored at −20°C. Assays were performed as previously described and β-galactosidase specific activity was expressed as Miller units mg^−1^ protein [66]. Protein concentrations were determined using the Bio-Rad protein assay (BioRad, Hercules, CA) [67]. All experiments were carried out in triplicate.

### Microarray experiments

RNA samples for tiling arrays were prepared as described above using cultures of *S. aureus* strains HG001 and ST1036 (Δ*graRS*) grown in TSB with 50 µg ml^−1^ colistin for GraSR induction, with an additional 2-fold dilution step in killing buffer (20 mM Tris-HCl pH 7.5, 5 mM MgCl_2_, 20 mM NaN_3_) before centrifugation. RNA samples were then treated using the RNA Clean-Up kit (Norgen Biotech Corp., Canada) according to the manufacturer's recommendations and eluted in 40 µl of RNAse-free water. RNA preparations were quantified using a spectrophotometer at 260 nm and quality was checked by electrophoregram analysis on a BioAnalyzer (Agilent).

The BaSysBio Sau T1 NimbleGen 385K array was designed with a total of 383,452 features using OligoWiz 2.0 [68], with long iso-thermal probes (45–65 nt) covering the entire genome of *Staphylococcus aureus* NCTC 8325 (CP000253.1; [69] in 18 nt intervals on each strand (Hanne Jarmer, Technical University of Denmark, Lyngby, Denmark, personal communication). Tiling array experiments were carried out with 20 µg of each RNA sample, sent to Roche NimbleGen (Madison, WI, USA) where it was labelled and hybridized to the BaSysBio Sau T1 chip using the BaSysBio protocol for strand-specific hybridization [70]. All tiling array experiments were performed in triplicate using RNA isolated from independent cultures.

For data analysis, an aggregated expression value was computed for each Genbank annotated CDS as the median log_2_ intensity of probes lying entirely within the corresponding region (Pierre Nicolas, MIG INRA Jouy-en Josas, personal communication). To control for possible cross-hybridization artefacts the sequence of each probe was BLAST-aligned against the whole chromosome sequence and probes with a SeqS value above the 1.5 cut-off were discarded (SeqS is 2 for a probe with two exact matches) [71].

Aggregated intensity values of the individual samples were normalized by median scaling using the Rosetta Resolver software (version 7.2.1, Rosetta Biosoftware). Statistical significance of differential expression between the wild type and the mutant strain was then evaluated using the *Z*-test (ArrayStat software package, GE Lifesciences). Differentially expressed genes were chosen with a ratio between the wild-type and mutant strain ≥ 1.8 and a P-value ≤ 3.5×10^−4^. The complete MIAME compliant microarray data set is available at the NIH Gene Expression Omnibus (GEO) database under record number GSE26016:

 (http://www.ncbi.nlm.nih.gov/geo/query/acc.cgi?token=vbcbxmyiykmqotq&acc=GSE26016).

### cDNA synthesis and qRT-PCRs

cDNAs were synthesized using the iScript cDNA synthesis kit (Bio-Rad, Hercules, CA) according to the manufacturer's recommendations, in a 20 µl final reaction volume containing 1 µg total RNA. For qRT-PCR experiments, amplicon primers were designed using the BEACON Designer 4.02 software (Premier Biosoft International, Palo Alto, CA) (see Table 5). Quantitative real-time PCRs (qRT-PCRs), critical threshold cycles (CT) and *n*-fold changes in transcript levels were performed and determined as previously described and normalized with respect to 16S rRNA whose levels did not vary under our experimental conditions [45]. All experiments were performed in triplicate.

### Oxidative stress

*S. aureus* strains were treated with paraquat (methylviologen-dichloride hydrate) or H_2_O_2_ (Sigma Aldrich) and growth was followed in a 96-well microtiter plate (100 µl culture volume). Bacterial cultures were grown in TSB, diluted to an OD _600 nm_ = 0.05 and used to inoculate wells containing TSB with or without 40 mM paraquat, or 0.004% H_2_O_2_. Plates were incubated for 14 h with vigorous shaking at 37°C in a Synergy 2 thermoregulated spectrophotometer plate reader using the Gen5™Microplate Software (BioTek Instruments Inc., Winooski, VT). All experiments were performed at least in duplicate.

### High temperature growth

The effect of high temperatures was observed on *S. aureus* strains ST1120 (pMK4-Pprot), ST1116 (Δ*graRS*-c; Δ*graRS* pMK4-Pprot*graR*) and ST1117 (Δ*graRS* pMK4-Pprot), on cells grown in TSB at 37°C and diluted to an OD _600 nm_ = 0.2. Cultures were serially diluted (10^−1^, 10^−2^, 10^−3^ and 10^−4^ fold) and 10 µl of each dilution was spotted onto TSA plates, which were dried for 10 min at room temperature and incubated at 37°C or 44°C for 48 h.

## Supporting Information

Figure S1 ***lacZ*** **fusion control experiment expression analysis (A) Expression of** ***tufA*** **'** ***-lacZ*** **is not induced by colistin.** Expression of the *tufA'-lacZ* fusion was measured in strain ST1189 (HG001 *tufA*'*-lacZ*) during mid-exponential growth at 37° C in TSB (grey bars) or after treatment with 200 µg ml^−1^ colistin (black bars). β-Galactosidase assays were performed as described in Materials and Methods. **(B) Indolicidin induces expression of the** ***vraFG*** **operon and** ***mprF*** **.** Expression of *vraF*'*-lacZ* and *mprF*'*-lacZ* fusions in *S. aureus* strain HG001 was measured during mid-exponential growth at 37°C in TSB (grey bars) or after treatment with 5 µg ml^−1^ indolicidin (black bars). β-Galactosidase assays were performed as described in *Experimental Procedures*.
(TIF)

Figure S2 **Linear correlation between microarray and qRT-PCR experiments for expression of GraSR-dependent genes.** Fold changes in expression as measured by qRT-PCR and transcriptome analysis measured for 4 representative genes in the *S. aureus* HG001 strain relative to the ST1036 (Δ*graRS*) strain grown in the same conditions were plotted against each other to evaluate their correlation. Data points were analyzed in triplicate by both methods.
(TIF)

Table S1 **Genes positively controlled by GraSR.**
(XLS)

Table S2 **Genes negatively controlled by GraSR.**
(XLS)
