# Supplementary figures and images for: Investigation of the Staphylococcus aureus GraSR Regulon Reveals Novel Links to Virulence, Stress Response and Cell Wall Signal Transduction Pathways
Source: PLoS One. 2011 Jul 1;6(7):e21323. doi: 10.1371/journal.pone.0021323 (PMC3128592; doi:10.1371/journal.pone.0021323)

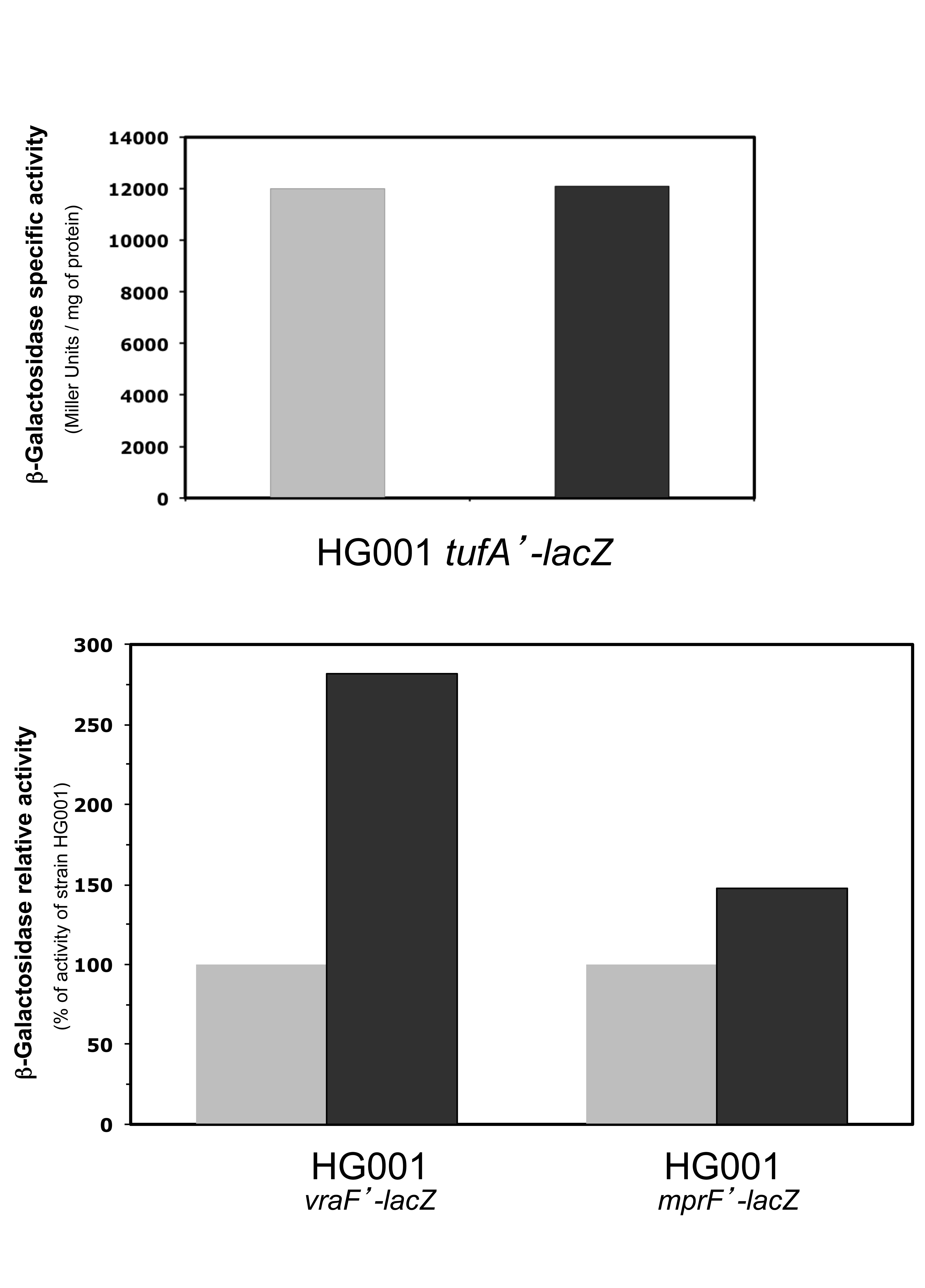

Supplement: Figure S1 — lacZ fusion control experiment expression analysis (A) Expression of tufA ' -lacZ is not induced by colistin. Expression of the tufA'-lacZ fusion was measured in strain ST1189 (HG001 tufA'-lacZ) during mid-exponential growth at 37° C in TSB (grey bars) or after treatment with 200 µg ml−1 colistin (black bars). β-Galactosidase assays were performed as described in Materials and Methods. (B) Indolicidin induces expression of the vraFG operon and mprF . Expression of vraF'-lacZ and mprF'-lacZ fusions in S. aureus strain HG001 was measured during mid-exponential growth at 37°C in TSB (grey bars) or after treatment with 5 µg ml−1 indolicidin (black bars). β-Galactosidase assays were performed as described in Experimental Procedures. (TIF) [file pone.0021323.s001.tif]

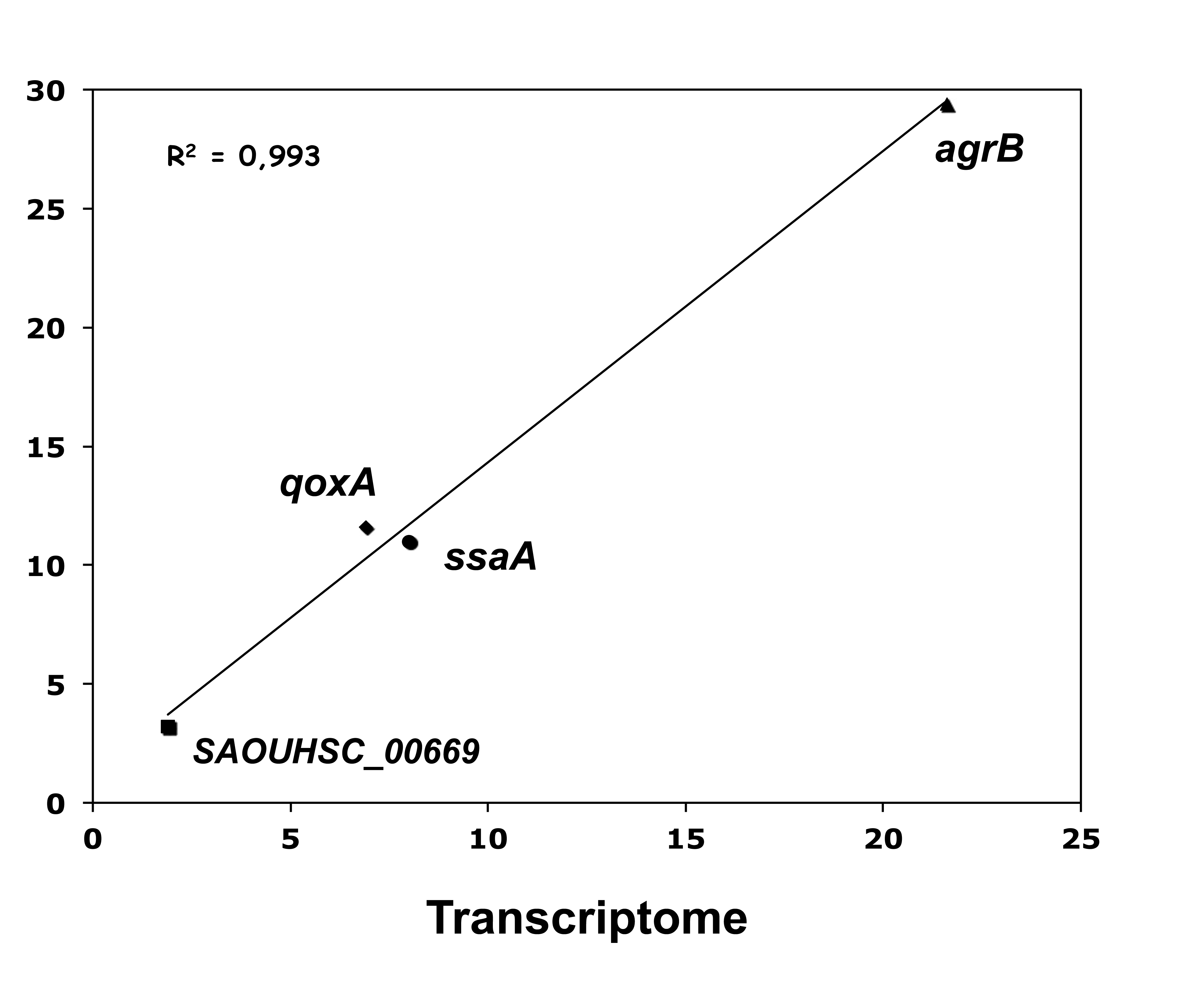

Supplement: Figure S2 — Linear correlation between microarray and qRT-PCR experiments for expression of GraSR-dependent genes. Fold changes in expression as measured by qRT-PCR and transcriptome analysis measured for 4 representative genes in the S. aureus HG001 strain relative to the ST1036 (ΔgraRS) strain grown in the same conditions were plotted against each other to evaluate their correlation. Data points were analyzed in triplicate by both methods. (TIF) [file pone.0021323.s002.tif]
